# Recovering Speech from Vibrations: Principles and Algorithms in Radar and Laser Sensing

**DOI:** 10.3390/s26082553

**Published:** 2026-04-21

**Authors:** Emily Bederov, Baruch Berdugo, Israel Cohen

**Affiliations:** Andrew and Erna Viterbi Faculty of Electrical & Computer Engineering, Technion–Israel Institute of Technology, Haifa 3200003, Israel; emilybederov@campus.technion.ac.il (E.B.); bberdugo55@gmail.com (B.B.)

**Keywords:** millimeter-wave radar, laser Doppler vibrometry, radar-based speech recognition, acoustic eavesdropping, non-acoustic sensing, speech reconstruction, non-line-of-sight sensing, deep learning, automatic speech recognition (ASR)

## Abstract

Sensing audio using non-acoustic modalities such as millimeter-wave radar and laser-based systems has emerged as an active research area with significant implications for privacy, security, and robust speech processing. These approaches recover speech-related information from vibration measurements captured by non-acoustic sensing modalities. Prior work spans a wide range of techniques, from classical signal-processing pipelines to modern machine-learning and deep-learning models, enabling applications such as speech reconstruction, eavesdropping, automatic speech recognition, and noise-robust enhancement. Some systems rely on radar or laser sensing as a standalone audio surrogate, while others fuse radar-derived features with microphone signals to improve robustness in noisy or non-line-of-sight environments. Experimental results across the literature demonstrate that recovering intelligible speech or discriminative speech features from radar or laser-sensed vibrations is feasible under controlled conditions. However, performance remains sensitive to practical factors including sensing distance, object material and geometries, environmental interference, multipath effects, and task complexity. Not all speech-related tasks are reliably solved, particularly in unconstrained real-world scenarios. Overall, the field is rapidly evolving, with open challenges in robustness, generalization, and deployment, offering several promising directions for future research.

## 1. Introduction

Human speech is conventionally captured using acoustic microphones; however, recent studies have shown that speech can also be sensed remotely through non-acoustic physical side channels. When speech is produced, it induces minute mechanical vibrations on the human body, electronic devices, and surrounding objects, which can be captured without direct acoustic access by exploiting alternative physical carriers such as radio-frequency signals or light. This capability enables speech sensing in scenarios where microphones fail, including through obstacles, in high-noise environments, or without line-of-sight. At the same time, such capabilities raise significant privacy and security concerns, as they enable covert eavesdropping on speech without the subject’s awareness [1,2].

Among the proposed non-acoustic approaches, millimeter-wave (mmWave) radar-based sensing and laser Doppler vibrometry (LDV) and related optical sensing methods have emerged as two dominant families of technologies. mmWave radar systems infer speech-induced vibrations by analyzing phase or frequency modulations in reflected radio signals, enabling operation in non-line-of-sight and through-wall scenarios [3,4,5]. In contrast, laser Doppler vibrometry (LDV) and optical methods recover speech by measuring vibration-induced changes in reflected coherent or ambient light, offering high displacement sensitivity and long sensing ranges under favorable line-of-sight conditions [6,7]. In some settings, speech information can also be recovered indirectly via vibrations of secondary reflective surfaces, even when the speech source itself is not in direct line of sight [8]. Despite relying on different physical carriers, both modalities ultimately address the same inverse problem: inferring speech content from bandwidth-limited, noise-sensitive vibration measurements.

**Figure 1:**
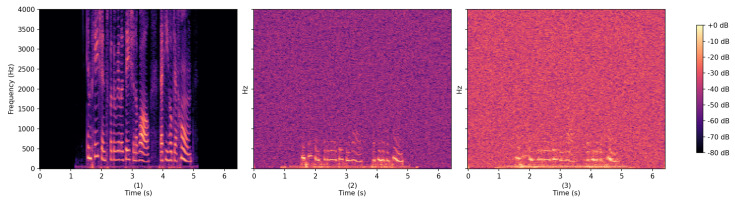
Example spectrograms of radar-sensed speech. (1) Clean speech spectrogram. (2) Radar measurement through soundproof glass and a diaphragm. (3) Radar measurement through soundproof glass and aluminum foil. Using the RASE Challenge dataset.

Research in this area has evolved rapidly from early proof-of-concept demonstrations to increasingly sophisticated signal-processing and deep-learning-based systems. Radar-based works have progressed from constrained keyword recognition and device-specific eavesdropping to unconstrained-vocabulary speech recognition and radar-only automatic speech recognition frameworks [5,9,10]. Similarly, laser-based approaches have advanced from classical vibrometry toward learning-assisted speech recognition using auxiliary LDV features [6,7]. Despite this progress, the performance of non-acoustic ASR systems remains substantially lower than that of microphone-based counterparts, particularly in robustness and generalization. As a result, the field remains far from closed and continues to offer a wide design space for advances in sensing hardware, signal processing, learning architectures, and cross-modal supervision. Across both modalities, fundamental challenges persist, including sensitivity to motion and multipath effects, strong dependence on object material and geometries, limited recovery of high-frequency speech components, and the lack of standardized datasets and evaluation protocols. Figure 1 presents an example radar spectrogram under representative conditions.

This review presents a unified, systematic examination of radar- and laser-based speech-sensing systems. Rather than treating the two sensing modalities independently, we analyze them within a common framework. We analyze prior work along shared dimensions including sensing physics, vibration targets, signal representations, learning architectures, achievable range, line-of-sight constraints, and privacy implications and organize the literature by both task objective (speech reconstruction, keyword recognition, and automatic speech recognition) and modeling paradigm (DSP-based methods, encoder–decoder networks, generative models, Transformer-based ASR, and multimodal fusion). This system-level perspective clarifies how underlying sensing constraints shape algorithmic and architectural design. It highlights key trade-offs across sensing modalities, including bandwidth limits, motion sensitivity, and target dependence. It also emphasizes the methodological distinction between reconstruction-driven pipelines and direct end-to-end ASR in terms of latency, fidelity, and deployability. Finally, the review identifies open challenges, including the lack of standardized datasets, limited cross-environment generalization, and the need for more physically grounded learning. It outlines promising directions for robust, privacy-aware non-acoustic speech sensing.

## 2. Physical Principles of Non-Acoustic Speech Sensing

### 2.1. Radar-Based Vibration Sensing

Millimeter-wave radar systems sense vibration by transmitting electromagnetic waves and analyzing phase and frequency variations in the reflected signal. At typical operating frequencies between 60 and 80 GHz, the corresponding wavelengths are on the order of a few millimeters, making the received signal phase highly sensitive to small changes in propagation distance. As a result, micrometer-scale (or smaller) surface displacements induced by speech can be detected through time-varying phase modulations of the radar return.

Most recent radar-based speech sensing systems employ frequency-modulated continuous-wave (FMCW) operation, in which the carrier frequency is linearly swept over time using short chirps. FMCW signaling enables reflections from different ranges to be separated while preserving coherent phase information. After range processing, the signal associated with a given reflecting surface can be modeled as a narrowband complex baseband signal whose phase varies over time. Speech-induced vibrations appear as low-amplitude, time-varying phase perturbations superimposed on this signal, providing a physical mechanism for recovering speech-related information from radar measurements.

### 2.2. Laser-Based Vibrometry

Laser-based vibrometry measures surface motion by illuminating a target with coherent optical radiation and analyzing the phase or frequency shift of the reflected light. Due to the extremely short optical wavelengths (on the order of hundreds of nanometers), laser vibrometers exhibit very high displacement sensitivity, enabling detection of nanometer-scale vibrations produced by acoustic speech. In typical laser Doppler vibrometry (LDV) systems, surface motion along the laser line of sight induces a Doppler frequency shift or phase modulation in the reflected signal, from which surface velocity or displacement can be inferred.

A fundamental characteristic of laser vibrometry is its sensitivity to surface scattering properties. When coherent light is reflected from a rough surface, interference among multiple scattered optical paths produces speckle, resulting in amplitude fluctuations and phase instability in the measured signal. Speckle effects can introduce signal fading and measurement variability that depend on surface material, orientation, and illumination geometry. Consequently, while laser-based systems offer high precision under favorable line-of-sight conditions, their performance is strongly influenced by optical surface properties. Despite using different physical carriers, both radar- and laser-based approaches ultimately encode speech information as vibration-induced phase modulations, motivating a unified system-level analysis of non-acoustic speech-sensing methods.

## 3. Non-Acoustic Speech Sensing Approaches

### Modeling Paradigms in Radar-Based Speech Sensing

Radar-based speech sensing systems employ several distinct modeling paradigms, each reflecting different assumptions about signal fidelity, available supervision, and task objectives. This subsection summarizes the main model families used across the literature and relates them to the reviewed works.

**Figure 2:**
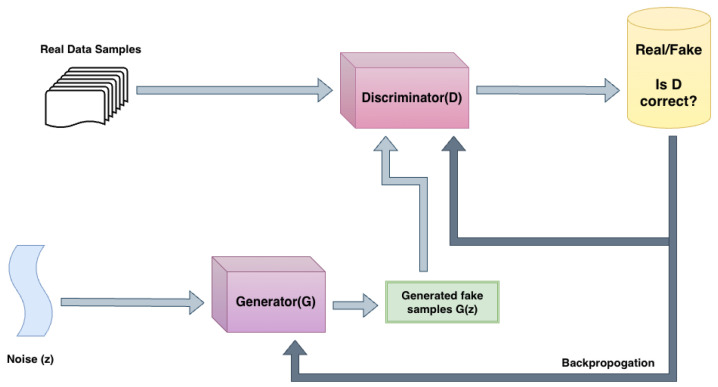
GAN Architecture.

**Generative Adversarial Networks (GANs):** GAN-based models (see Figure 2) are predominantly used for speech reconstruction, where the objective is to recover intelligible audio from severely band-limited, noisy radar measurements. In such settings, radar signals lack high-frequency components essential for speech intelligibility, rendering deterministic reconstruction approaches ineffective. A GAN is a natural choice for this modality because it treats vibration sensing as an image-to-image or signal-to-signal translation problem, in which the network must “fill in” spectral gaps introduced by the target material’s mechanical filtering.

A GAN framework consists of a generator that maps lossy radar-derived representations to audio or spectrogram estimates, and a discriminator that distinguishes reconstructed outputs from real speech samples. Through adversarial training, the generator learns to produce perceptually plausible speech by leveraging learned speech priors. The reasoning for using an adversarial objective rather than a simple Mean Squared Error (MSE) loss is that MSE often produces “blurry” spectrograms and muffled audio; the discriminator forces the generator to recover sharp, high-frequency harmonics that characterize natural human speech.

To improve perceptual quality, many systems adopt multi-resolution and multi-period discriminator designs, which jointly enforce spectral consistency and temporal realism across multiple time scales [11]. This paradigm is widely used in radar-based speech reconstruction systems such as mmSpeech, MilliEar, Radio2Speech, and EveGuard, where the emphasis is on perceptual intelligibility rather than strict physical signal fidelity. Variants and adaptations of the GAN framework, including CycleGAN-style unpaired translation and waveform-level generators such as WaveGAN, have also been explored in related contexts for audio generation and enhancement [12,13]. CycleGAN variants are particularly relevant here as they allow the model to learn the mapping between vibration and acoustic domains, even when perfectly synchronized, paired training data is unavailable, a common challenge in field-deployed radar systems.

A fundamental limitation of GAN-based reconstruction is the potential to hallucinate speech content that is not physically present in the sensed radar signal, as the generator is optimized for perceptual plausibility rather than for faithfully recovering missing acoustic information.

**Figure 3:**
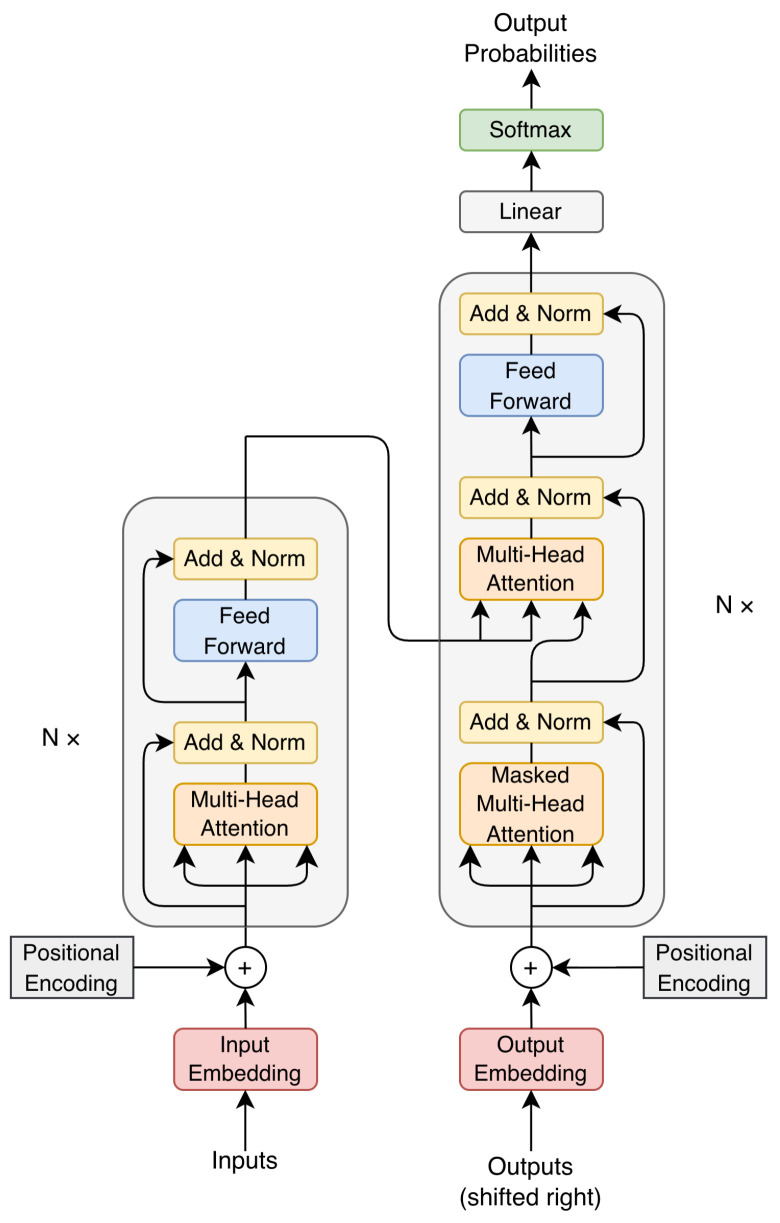
Transformer Architecture.

**Transformer-Based Models:** Transformer architectures (see Figure 3) are predominantly employed when the objective shifts from waveform reconstruction toward linguistic inference, most notably in automatic speech recognition. By modeling long-range temporal dependencies through self-attention, Transformers rely heavily on learned linguistic and contextual priors rather than precise signal fidelity. This property is particularly relevant for radar-based ASR, where sensed signals preserve coarse prosodic and rhythmic information but lack fine-grained phonetic detail.

The rationale for the “data-hungry” Transformer paradigm in this field lies in its inherent ability to map connections across highly distorted sequences. Non-acoustic signals suffer from “bursty” noise (due to sudden motion or multipath fading). The self-attention mechanism is a natural fit; it allows the model to dynamically “attend” to reliable signal frames while ignoring those buried in noise. By leveraging multi-head attention, the model can simultaneously track multiple rhythmic and phonetic features over time, effectively “re-stitching” the broken speech signal into a coherent linguistic representation.

Recent systems adopt Transformer-based architectures, such as Conformer, Whisper, and large-scale pretrained models. In these approaches, attention mechanisms compensate for weak or distorted acoustic cues by emphasizing linguistic consistency, contextual prediction, and temporal alignment, effectively shifting the modeling burden from signal accuracy to language modeling capability [14,15,16].

**Figure 4:**
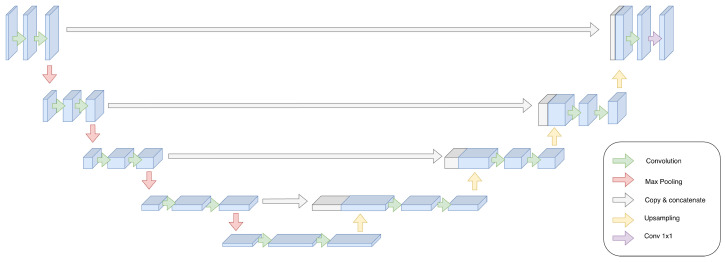
Unet Architecture.

**Encoder–Decoder and U-Net Architectures:** U-Net-style encoder–decoder networks (see Figure 4) are widely used for denoising and bandwidth expansion in the time–frequency domain. These models downsample the input spectrogram to extract high-level features and then upsample it to reconstruct the output, with skip connections preserving fine-grained temporal and spectral structure.

The rationale for adopting the U-Net paradigm in vibration sensing stems from its dual-domain learning capability. By projecting the radar signal into a downsampled latent space, the network can extract high-level ”structural” (spatial) features while filtering out localized measurement noise. The subsequent upsampling path then “reconstructs” the speech signal by mapping these features back to the time–frequency grid. Crucially, the skip connections provide an “alignment-preserving” mechanism, allowing the network to carry original temporal cues directly from the encoder to the decoder. This ensures that the speaker’s fine-grained rhythmic timing is preserved, preventing the temporal “blurring” that often occurs in standard autoencoders when processing weak radar returns.

Unlike GANs, U-Nets are typically trained with supervised reconstruction losses and do not explicitly enforce perceptual realism. RadioMic (RANet) and Radio2Speech employ U-Net variants to enhance radar-derived spectrograms, benefiting from the alignment-preserving properties of skip connections. While effective for structured noise removal, U-Nets alone are limited in their ability to recover missing high-frequency content without additional generative constraints.

**Multimodal Fusion Networks:** Several systems treat radar as a complementary modality rather than a standalone speech sensor. In these approaches, radar provides robustness to acoustic noise and occlusion, while microphones supply rich spectral detail. The rationale for this fusion is the “Complementary Physics” of the two sensors: microphones are sensitive to air pressure but fail in high-noise environments, whereas radar is immune to acoustic noise but lacks spectral resolution. By fusing them, the network can use the radar as a “gate” or “guide” to focus on the speaker’s voice while ignoring environmental noise. WaVoice employs attention-based fusion with SENet-style inter-attention to combine radar and audio features for robust ASR. mmMIC integrates radar embeddings into conventional ASR pipelines to stabilize recognition under adverse conditions. RadioSES uses radar cues to guide speech enhancement and separation by informing speaker counting and localization. These fusion-based models do not attempt to reconstruct speech from radar alone; instead, they use radar to constrain and regularize audio processing.

**Discriminative Models for Identity and Security:** In contrast to reconstruction and recognition systems, identity-oriented approaches focus on extracting speaker-specific features rather than speech content. WavoID employs discriminative embedding networks trained to separate speakers based on radar-captured vibration patterns. The decision to focus on discriminative embeddings rather than reconstruction is often driven by “Privacy-by-Design.” By mapping vibrations directly to a unique speaker ID in a latent space, the system can perform biometric authentication without ever reconstructing the actual spoken words, thereby mitigating the risk of sensitive information leakage. These systems avoid speech reconstruction entirely, reducing privacy leakage while exploiting the correlation between vocal dynamics and radar-measured vibrations.

**Classical Digital Signal Processing (DSP):** A small number of works rely solely on traditional signal processing without learning-based components. mmProcess and UWHear extract speech-related information using range processing, phase unwrapping, filtering, and spectral analysis. These approaches are maintained in the literature because they provide “Zero-Shot” interpretability. Unlike black-box neural networks, DSP methods do not require massive datasets and serve as the physical foundation for the field; they are essential for real-world scenarios where the computational latency of a GAN or Transformer is prohibitive. These approaches offer transparency and do not require training data, but they are fundamentally limited by noise, multipath effects, and their inability to recover missing spectral content. As such, DSP-based methods typically recover only coarse prosodic structure rather than intelligible speech and serve as important baselines that highlight the limits of learning-free radar speech sensing.

## 4. Radar-Based Sensing

**Figure 5:**
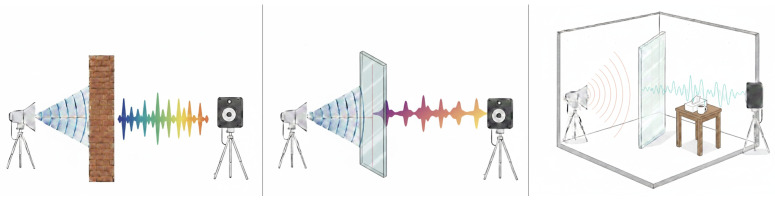
(1) is a sensing scenario with a brick wall, (2) sensing scenario with soundproof glass wall, (3) is a sensing scenario with both soundproof glass wall and objects blocking the speech signal.

Radar-based speech sensing research investigates how millimeter-wave (mmWave) and radio-frequency (RF) signals can be used to recover speech-related information by sensing vibration-induced modulations on objects coupled to sound sources [1,2,17]. Across the literature, existing systems can be broadly understood through their primary objective: (i) radar-only eavesdropping and speech reconstruction, (ii) radar-as-a-microphone and bandwidth expansion, (iii) direct radar-based automatic speech recognition, (iv) multimodal radar-audio fusion for robustness, and (v) privacy-aware analysis and defenses [1,2,9,17,18,19]. While these categories overlap in sensing principles, they reflect distinct modeling choices and design trade-offs., (Figure 5) illustrates sensing configuration scenarios.

**Radar-only eavesdropping and vibration-based speech recovery:** Early work focused on establishing whether intelligible speech can be recovered using radar alone, without any acoustic sensor near the target [1]. mmSpy demonstrates that by tracking a stable reflection point near a smartphone earpiece and extracting phase variations from a single radar range bin, speech-induced vibrations can be captured as an audio-like signal sampled at the radar chirp repetition rate [1]. In the reported configuration, phase is sampled at approximately 12.5 kHz and downsampled to 8 kHz, resulting in an effective vibration bandwidth of 0–4 kHz [1]. After spectrogram conversion, denoising with a convolutional encoder–decoder network, and waveform reconstruction using Griffin–Lim, the system achieves intelligible recovery of short utterances and high accuracy on digit and keyword recognition tasks under line-of-sight conditions [1]. However, mmSpy also exposes fundamental limitations of radar vibrometry: performance degrades rapidly with distance, multipath interference, and motion, and the recoverable signal is severely band-limited compared to microphone audio [1]. Wavesdropper studies a complementary threat model focused on through-wall word detection rather than high-fidelity waveform reconstruction [20]. By leveraging commercial mmWave devices to sense speech-related vibration cues and directly classify spoken content, Wavesdropper reports high accuracy on a fixed-size vocabulary (57 words) in controlled evaluations, emphasizing robustness to occlusion at the cost of unconstrained speech recovery [20].

UWHear explores a related eavesdropping problem but departs from narrowband FMCW sensing by using impulse radio ultra-wideband (IR-UWB) transmissions to target through-wall scenarios [21]. By exploiting wideband wireless reflections, UWHear demonstrates the recovery and separation of multiple sound sources behind walls using a learning-free signal-processing pipeline based on time-of-flight estimation, vibration extraction, and source separation [21]. This work highlights that walls and structural elements can act as unintended vibration sensors and shows that performance is highly dependent on wall material, geometry, and environmental clutter. That robustness degrades in dynamic settings [21]. mmEavesdropper targets directional eavesdropping with mmWave radar by introducing signal-augmentation and calibration strategies that enhance micro-vibration extraction under practical sensing distortions [22]. In contrast to purely feasibility-oriented pipelines, it explicitly addresses directionality and the weakness of vibration signals through modeling and augmentation, demonstrating improved recovery under more challenging configurations [22].

Building on these feasibility studies, We Can Hear You (mmSpeech) addresses the limited vocabulary and poor intelligibility of earlier approaches by introducing an end-to-end learning-based radar eavesdropping framework [2]. The system captures loudspeaker-induced surface vibrations using FMCW mmWave radar. It emphasizes careful selection of vibrating materials, such as PET films, to maximize vibration fidelity under both line-of-sight and through-wall conditions. A generative adversarial network is employed to refine noisy vibration spectrograms and infer missing frequency components, with a discriminator combining multi-period and multi-resolution structures to enforce perceptual realism. Experimental results show substantially improved intelligibility and objective speech quality metrics, as well as generalization to unseen speakers. At the same time, mmSpeech demonstrates that leakage remains concentrated in the 0–4 kHz band, and that masking low-frequency vibrations can significantly suppress recovery, underscoring the strong dependence on surface material and placement.

mmPhone further expands the radar eavesdropping threat model by exploiting piezoelectric materials to enhance vibration sensitivity [23]. Instead of relying solely on naturally vibrating surfaces, mmPhone uses a PVDF piezoelectric film as a passive transducer whose electromagnetic reflection properties vary with sound-induced charge. These variations are sensed by a 77–81 GHz FMCW radar, processed to extract pitch and harmonic structure, and then used for waveform synthesis. This design enables intelligible speech recovery even in soundproofed environments where surface vibrations are weak, significantly broadening the range of feasible attack scenarios. Nevertheless, the approach requires the presence or placement of a suitable piezoelectric film and remains sensitive to multipath and phase noise. mmEcho further strengthens the radar-only eavesdropping line by proposing a mmWave-based acoustic eavesdropping method that emphasizes precise vibration extraction (including phase alignment) to enable robust audio reconstruction across varied positions, orientations, and real-world conditions [24]. This framing complements learning-based systems by highlighting that careful signal processing can substantially improve vibration recovery without relying exclusively on large end-to-end training pipelines. Beyond loudspeakers and passive vibrating surfaces, mmEve demonstrates an end-to-end eavesdropping attack that recovers speech emitted from a smartphone earpiece using a commercial mmWave radar, explicitly targeting “confidential” call scenarios and motion resilience [25]. mmEar introduces the same privacy risk to headphones by demonstrating the feasibility of mmWave eavesdropping on headphone playback, pushing sensitivity limits in low-vibration settings, and broadening the set of everyday devices that can leak speech through vibration channels [26].

**Diffusion-Based Generative Reconstruction Models:** Diffusion-based reconstruction has been explored in the paper mmWave radar study, mmSound [27], which applies conditional diffusion models to recover high-frequency speech components lost during radar-based vibration sensing. By iteratively refining radar-derived signals toward a learned speech distribution, diffusion enables perceptually high-quality broadband reconstruction from severely band-limited inputs. However, this capability relies heavily on strong generative priors, which increases the risk of hallucinating speech content not directly supported by physical measurements. Moreover, the iterative inference process introduces substantial computational cost and latency, limiting suitability for real-time or streaming applications. Consequently, diffusion-based radar-to-speech reconstruction is best viewed as an upper bound on perceptual plausibility rather than a physically faithful or deployment-oriented solution.

**Radar-as-a-microphone and bandwidth expansion:** Beyond direct eavesdropping, several works explicitly conceptualize radar as a microphone-like sensing modality and focus on compensating for the fundamental bandwidth and noise limitations of RF-based vibrometry [5,17]. MilliEar proposes a two-stage pipeline that combines range–Doppler-based spectrogram extraction with conditional GAN-based audio reconstruction from paired radar and audio data [5]. RadioMic formulates RF acoustic sensing as a joint denoising and bandwidth expansion problem and introduces RANet, a U-Net-like autoencoder operating on time–frequency spectrogram representations [17]. By reconstructing missing high-frequency components from low-frequency RF observations, these systems achieve improved perceptual quality and intelligibility compared with classical signal-processing baselines. However, both works emphasize that bandwidth expansion relies on learned speech priors and may hallucinate content, and that performance remains highly sensitive to environmental conditions, multipath, and surface reflectivity.

Radio2Speech continues this line of work by focusing on high-quality speech reconstruction using deep generative models [28]. A radar-to-speech U-Net maps radar spectrograms to audio spectrograms, followed by waveform reconstruction with a neural vocoder. Trained end-to-end on paired radar-audio data with spectral and waveform-level losses, the system produces substantially more intelligible speech than earlier RF-based approaches. At the same time, reliance on paired training data and degradation under severe multipath or poor vibration quality highlight ongoing generalization challenges.

**Direct radar-based automatic speech recognition:** Rather than reconstructing audio waveforms, ref. [9] shifts focus to direct radar-based ASR. The system captures speech-induced vibrations using mmWave radar and converts them into feature sequences, which are then processed by a streaming Transformer architecture. A chunk-wise encoder and CTC-triggered attention decoder ensure low-latency inference suitable for real-time use. To mitigate the modality gap between radar and audio, Radio2Text introduces cross-modal knowledge distillation that aligns attention maps, hidden states, and output distributions between an audio teacher network and a radar student network. This distillation strategy significantly improves recognition accuracy over radar-only baselines and enables unconstrained-vocabulary ASR. However, performance remains below that of microphone-based systems and is sensitive to radar configuration and environment. Moreover, mmWave-Whisper leverages synthetic radar–speech pairs to fine-tune selected components of a pretrained Whisper model, enabling the network to adapt its linguistic representations to radar-derived inputs [10]. Rather than retraining the model end-to-end, this approach leverages Whisper’s strong language priors while learning a radar-specific front-end alignment, enabling direct radar-to-text transcription despite the lack of fine-grained acoustic detail.

**Multimodal radar audio fusion for robustness:** Several systems treat radar not as a replacement for microphones, but as a complementary sensing modality that improves robustness in challenging acoustic conditions [3,18,29,30]. mmMIC combines mmWave radar measurements with microphone audio using a multimodal neural network that fuses vibration-derived and acoustic features, reporting improved ASR performance under noise and occlusion compared to audio-only baselines [29]. WaveEar similarly leverages mmWave sensing but focuses on near-throat measurements to directly capture vocal-fold vibrations [30]. By mapping reflected signals to speech spectrograms using an encoder–decoder network, WaveEar demonstrates improved robustness to ambient noise compared with far-field sensing, while remaining sensitive to throat localization and head motion.

AmbiEar extends multimodal and radar-only concepts to non-line-of-sight scenarios by treating the environment itself as a distributed set of passive sensors [3]. The system aggregates vibration data from multiple reflectors via signal superposition and filtering, then uses an encoder–decoder network for voice recognition. Experiments show robust recognition accuracy under occlusion, although performance depends strongly on reflective geometry and stable multipath. RadioSES applies radar–audio fusion to speech enhancement and separation, using mmWave radar to estimate speaker presence, number of speakers, and spatial cues that guide a deep audio–radio fusion network optimized with scale-invariant SDR objectives [18]. Results demonstrate consistent improvements over audio-only baselines and reveal sensitivity to localization errors and environmental geometry.

**Identification, classical processing, and defenses:** Beyond recovering speech content, radar-based vibration sensing has been explored for speaker identification and security-related tasks [31]. WavoID uses mmWave radar to capture voice-induced vibrations and employs a CNN-based embedding network to extract speaker-dependent features for classification, achieving high identification accuracy under noisy conditions compared to audio-only systems, albeit requiring enrollment data and not supporting ASR.

At the same time, mmProcess demonstrates that learning-free signal-processing pipelines based on range processing, phase unwrapping, adaptive filtering, and spectral enhancement can directly recover coarse speech structure, such as rhythm and pitch, from radar phase measurements [32]. However, transparent and independent of training data, intelligibility remains significantly lower than that of deep learning approaches and is sensitive to noise and multipath. Finally, EveGuard addresses the growing privacy concerns associated with vibration-based side-channel leakage by introducing an active defense mechanism [19]. By injecting imperceptible low-frequency perturbations into audio signals using a perturbation generator and a domain translation network, EveGuard significantly degrades the quality of reconstructed audio and ASR accuracy for eavesdroppers while preserving perceptual quality for human listeners. However, its effectiveness depends on attacker assumptions and does not protect against direct throat-vibration sensing.

**End-to-end ASR vs. reconstruction followed by ASR fine-tuning:** Beyond data availability and sensing-bandwidth limitations, the choice between reconstruction-based pipelines and end-to-end (E2E) ASR has important computational and architectural implications [2,5,9,17,28]. Reconstruction-based approaches typically rely on generative models, most commonly GANs or diffusion-inspired networks, to synthesize acoustic waveforms or spectrograms from non-acoustic measurements. While such models can improve perceptual quality, they are computationally expensive, introduce substantial inference latency, and are poorly suited for real-time or streaming operation. Moreover, despite producing an audio-like signal, these pipelines still require downstream ASR fine-tuning to address reconstruction artifacts and domain mismatch, thereby shifting the learning challenge rather than eliminating it. From this perspective, reconstruction followed by ASR constitutes an indirect solution to a fundamentally recognition-driven problem.

In contrast, end-to-end ASR systems optimize the target objective directly by mapping non-acoustic features to linguistic units, bypassing explicit audio synthesis and reducing both model complexity and latency. Although E2E approaches remain dependent on large-scale supervision or cross-modal teacher–student distillation, they offer a more principled, task-aligned path to efficient speech recognition from non-acoustic sensors. More broadly, this distinction highlights a second critical design axis beyond sensing modality: whether learning is driven by perceptual reconstruction or by recognition itself. Reconstruction-based pipelines prioritize audio plausibility under severe bandwidth and noise constraints, often relying on learned priors to hallucinate missing high-frequency content. In contrast, direct ASR pipelines treat non-acoustic sensing as a noisy communication channel to language and optimize transcription accuracy directly.

### Key Challenges and Open Problems

Despite rapid progress in radar-based speech sensing, the field faces several fundamental challenges that limit scalability, reproducibility, and real-world deployment [1,2,3,9,17,18,19]. These challenges arise from a combination of data scarcity, physical sensing constraints, modeling assumptions, and evaluation practices.

A primary bottleneck across most radar-based speech-sensing systems is the lack of large-scale, standardized datasets. Collecting paired radar and audio data is technically complex, time-consuming, and highly sensitive to experimental setup, including radar configuration, surface material, geometry, and environmental conditions. As a result, most existing works rely on small, custom datasets captured under tightly controlled scenarios. This data scarcity complicates supervised training, limits model capacity, and increases the risk of overfitting to specific environments or sensing configurations. Moreover, the absence of publicly available benchmark datasets makes fair comparison across methods difficult and hinders systematic progress in the field.

Recent initiatives, such as the Radar-based Acoustic Sensing and Eavesdropping (RASE) challenge at ICASSP 2026 [33], highlight the growing interest in radar-based speech sensing and mark a turning point for the community by providing a two-track benchmark collected with a TI AWR2243BOOST mmWave FMCW radar through glass walls. Figure 1 illustrates example spectrograms of radar signals across different sensing scenarios. The challenge defines two distinct data scenarios that represent the primary physical hurdles in the field:

- **Task 1: Direct Diaphragm Vibration (Simple Case):** The radar captures strong vibrations directly from a loudspeaker diaphragm. While the resulting radar spectrogram exhibits visible speech content, it remains band-limited and noisier compared to the microphone reference.
- **Task 2: Secondary Surface Vibration (Challenging Case):** The radar senses vibrations from a secondary surface (e.g., thin aluminum foil) placed near the loudspeaker. In this scenario, speech components are often nearly buried in the noise floor, posing a high-difficulty task of recovering speech from weak, indirect surface modulations.

While these tasks provide a curated dataset of paired radar and microphone recordings, a significant limitation remains: the RASE data is provided as pre-processed waveforms. This abstraction prevents researchers from optimizing the low-level radar front end, such as phase unwrapping or range-Doppler estimation. To achieve true robustness, future datasets must bridge this gap by providing raw I/Q sensor data across a greater diversity of material-specific vibration signatures and environmental signal-to-noise ratios (SNRs).

Furthermore, the scale of the RASE dataset (approximately 10 h) highlights a significant challenge in non-acoustic speech research: the “data-hunger” of modern architectures versus the high cost of specialized data collection. A 10-h corpus is generally insufficient for training end-to-end generative models from scratch without risking severe overfitting to the specific acoustic and material conditions of the glass-wall setup.

To overcome these barriers, future efforts must move toward **task-specific multi-domain datasets**. We identify three critical requirements for the next generation of non-acoustic data:

1. **Raw Modality Integrity:** Datasets must include raw I/Q radar chirps and complex-spectrum laser returns to allow for the optimization of low-level signal processing alongside high-level machine learning.
2. **Task-Agnostic Diversity:** Different tasks require distinct data types. For example, *Keyword Spotting (KWS)* requires massive temporal variety and precise labels of word occurrences, whereas *Automatic Speech Recognition (ASR)* needs hundreds of hours of data and transcriptions for overall convergence. In multi-speaker scenarios, there is an additional need for diverse spatial and spectral variations.
3. **Environmental and Material Variance:** To prevent overfitting, datasets must explicitly label the vibration target (e.g., PET film vs. drywall) and account for various setups, including different distances and physical barriers.

Beyond the data’s physical diversity, the community must establish **Standardized Metadata and Accessibility Protocols**. To ensure that published datasets are truly interoperable, every non-acoustic dataset should be accompanied by a “Sensing Context” manifest containing:

1. **Hardware Configuration:** Documentation of the sensor’s operating parameters (e.g., radar chirp slope and sampling rate) to allow for signal normalization across different device architectures.
2. **Geometric Ground Truth:** Precise measurements of the sensing distance, grazing angle, and sensor-to-target alignment. This is critical because vibration amplitude decays non-linearly with distance and angle.
3. **Target Material Physics:** Rather than qualitative labels like “foil,” datasets should provide quantitative properties such as surface density and material thickness, which dictate the mechanical resonance frequencies of the target.
4. **Temporal Alignment:** Verification of sub-millisecond synchronization between the vibration sensor and the reference microphone to ensure that phase-aware models can accurately map non-acoustic features to acoustic ground truth.

By publishing under these standardized circumstances, the community can move toward a **Universal Non-Acoustic Pre-training** paradigm. This would allow researchers to pool small-scale datasets into a singular, massive multi-site repository, providing the diversity needed to solve complex multi-speaker separation and long-distance sensing challenges.

Beyond data availability, radar-based speech sensing is fundamentally constrained by the physical limitations of vibration-based sensing. Millimeter-wave radars capture surface displacements rather than acoustic pressure waves, and vibration amplitudes decay rapidly with frequency. Consequently, the observable bandwidth is typically limited to low-frequency components below approximately 3–4 kHz, even under favorable conditions. Learning-based reconstruction models attempt to compensate for this limitation by inferring or hallucinating missing high-frequency content using speech priors [2,5,17,28]. While this can improve perceptual quality and intelligibility, it introduces ambiguity and raises questions about signal fidelity, authenticity, and generalization. In particular, reconstructed high-frequency components are not physically measured and may diverge significantly from the true speech signal, especially in unseen acoustic or linguistic contexts.

These physical constraints also interact with distance and geometry in nontrivial ways [1,2,17]. Radar sensitivity to micro-vibrations decreases rapidly with range, and phase measurements become increasingly corrupted by multipath interference, motion, and oscillator noise at longer distances. Many existing systems demonstrate promising results only at short ranges or under carefully aligned line-of-sight conditions, while performance degrades sharply with increasing distance or when reflections become unstable. Robust long-range sensing remains an open problem, particularly in realistic indoor environments with dynamic clutter and human motion.

For automatic speech recognition, an additional challenge lies in evaluation methodology and benchmarking. However, most studies evaluate ASR performance on proprietary datasets without aligning to standard ASR benchmarks or protocols. As a result, it is difficult to assess how radar-based ASR systems compare with established audio-based models under comparable conditions, or to quantify the true benefit of distillation relative to language-modeling capacity. The lack of standardized benchmarks for radar-based ASR limits reproducibility and obscures the relationship between sensing quality and linguistic performance.

Another open question concerns architectural design choices, particularly the common pipeline of speech reconstruction followed by ASR [5,9,17,28]. Many systems first attempt to reconstruct an audio waveform from radar measurements and then apply a conventional ASR model. While intuitive, this two-stage approach compounds errors: artifacts introduced during reconstruction may degrade downstream recognition, and reconstruction objectives do not necessarily align with recognition performance. Direct radar-to-text models challenge this paradigm by bypassing waveform reconstruction entirely, but the conditions under which each approach is preferable remain poorly understood. A principled comparison between reconstruction-based and direct recognition pipelines, grounded in both physical sensing constraints and linguistic modeling, is still lacking.

Multimodal fusion introduces its own challenges. While combining radar with microphones can significantly improve robustness under noise or occlusion, fusion performance depends heavily on synchronization, calibration, and environmental geometry. Errors in radar-based localization or speaker association can degrade performance rather than improve it, and the optimal fusion strategy may vary across tasks such as recognition, enhancement, and separation. Designing fusion architectures that are robust, interpretable, and transferable across environments remains an open research problem.

Finally, growing awareness of privacy risks introduces unresolved questions at the intersection of sensing, learning, and defense. Radar-based speech sensing demonstrates that speech content can leak through unintended physical channels, even in acoustically isolated environments. Recent eavesdropping systems further show that this leakage can extend to common everyday endpoints such as smartphone earpieces and headphones, reinforcing the practical privacy risk surface of vibration-based sensing. Defensive mechanisms such as adversarial perturbations show promise, but their effectiveness depends on assumptions about attacker models and sensing modalities, and they may not generalize to all vibration-based sensors. Understanding the fundamental limits of both attack and defense and developing sensing-aware privacy guarantees remain important and largely unexplored directions.

Addressing these challenges will require coordinated efforts in dataset creation, standardized evaluation protocols, physically grounded modeling, and integrated consideration of both sensing capabilities and privacy implications. Without such advances, radar-based speech sensing is likely to remain confined to narrow experimental settings rather than achieving robust real-world deployment.

## 5. Laser and Optical Speech Sensing

### 5.1. Scope and Review

Laser-based speech sensing comprises a class of remote acoustic acquisition techniques in which speech-induced mechanical vibrations are measured optically rather than acoustically. In contrast to conventional microphones, these systems do not directly sense air-pressure fluctuations; instead, they infer speech information from the interaction between laser illumination and vibrating surfaces. This review is deliberately restricted to three laser-based modalities: Laser Doppler Vibrometry (LDV), laser microphones, and LiDAR-derived acoustic sensing. Other optical approaches that do not rely on laser illumination, such as purely vision-based systems, are excluded except where conceptual contrast is required. Within this scope, the literature is organized according to the physical quantity measured by the laser system, the signal representation derived from that measurement, and the class of speech-related tasks enabled by the resulting signal.

**Figure 6:**
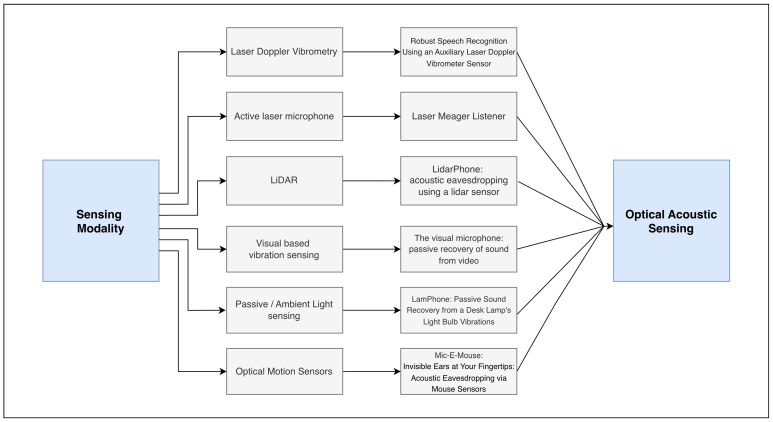
Overview of laser-based sensing modalities.

There exist multiple laser-based sensing modalities in Figure 6, while this work focuses only the mose representative modalities relevant to speech enhancement.

### 5.2. Laser Doppler Vibrometry

#### 5.2.1. Measurement Principle and Signal Characteristics

Laser Doppler Vibrometry measures the surface velocity of a vibrating object by detecting the Doppler shift of a reflected laser beam. When a surface is excited by speech, its vibration velocity encodes low- to mid-frequency components of the acoustic signal. Importantly, the LDV output is not a pressure waveform but a mechanically filtered representation whose spectral characteristics depend strongly on the object’s material properties, geometry, and boundary conditions.

As a consequence, LDV-based speech signals exhibit object-dependent frequency responses, phase distortions, and missing spectral components, particularly at higher frequencies. These properties fundamentally distinguish LDV signals from microphone recordings and motivate specialized processing strategies.

#### 5.2.2. Classical LDV Speech Measurement and Enhancement

**Figure 7:**
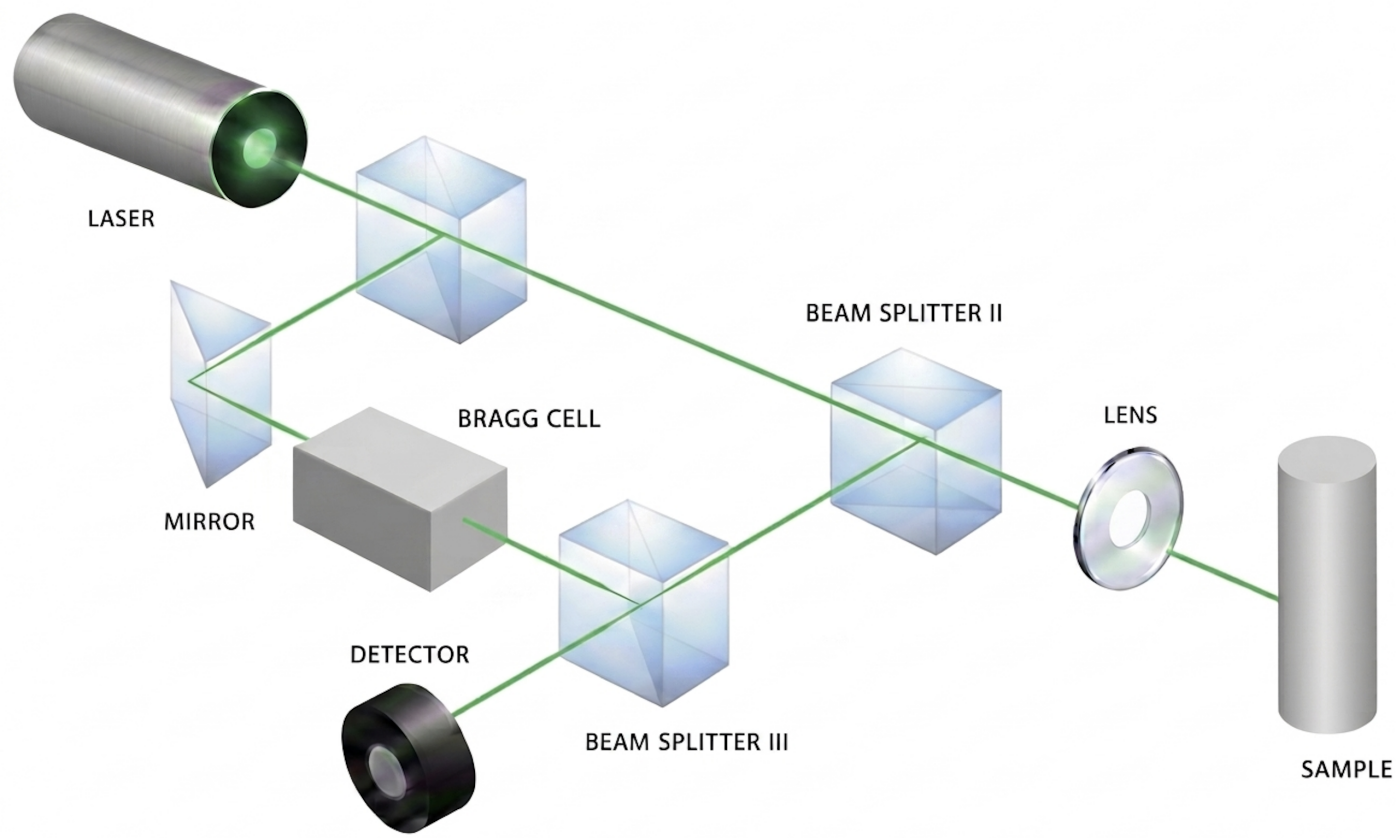
Laser Doppler vibrometer.

Early work on LDV-based speech sensing focused on demonstrating the feasibility of recovering intelligible speech from vibration measurements using classical digital signal processing techniques [34]. Typical approaches apply band-pass filtering, Wiener filtering, or spectral subtraction to suppress measurement noise and emphasize speech-dominant frequency bands. Although such methods improve intelligibility, they do not address the fundamental limitation of LDV sensing (LDV setup can be seen in Figure 7): the non-flat, object-specific mechanical transfer function. Consequently, classical enhancement techniques cannot restore missing frequency components or correct phase distortions, leaving speech signals perceptually degraded.

#### 5.2.3. LDV as an Auxiliary Modality for Robust ASR

A separate research direction treats LDV not as a standalone speech sensor, but as an auxiliary modality for automatic speech recognition. In this paradigm, features extracted from LDV signals are fused with conventional acoustic features to improve robustness under adverse acoustic conditions [7,35]. Because LDV measurements are largely immune to airborne acoustic noise, they provide complementary information when microphone signals are corrupted by such noise. However, these systems do not aim to reconstruct perceptually faithful speech waveforms. Instead, LDV features serve as noise-robust cues for phonetic discrimination. Several studies further propose regression models that map acoustic features to pseudo-LDV features, enabling large-scale ASR training without extensive LDV data collection [36]. While effective for recognition, this approach bypasses the physical sensing process and does not yield physically meaningful LDV signals.

#### 5.2.4. Deep Learning-Based LDV Speech Enhancement

More recent work applies deep learning to LDV speech enhancement, overcoming the limitations of classical DSP. These methods typically operate in the spectral domain and learn mappings from distorted LDV spectrograms to clean speech representations [37]. Common strategies include multi-stage networks that address noise suppression and spectral reconstruction separately, as well as frequency-band-specific models that infer missing high-frequency components from more reliable low-frequency information. Although these approaches significantly improve objective and subjective speech quality, their performance remains strongly tied to the mechanical characteristics of the training objects, and generalization across surfaces remains an open challenge.

### 5.3. Laser Microphones

#### 5.3.1. Classical Laser Microphone Systems

Laser microphones recover speech by measuring optical phase or intensity modulation caused by sound-induced vibrations of reflective surfaces, such as windows or walls [38]. Unlike LDV, which explicitly measures surface velocity, many laser microphone systems demodulate displacement-related phase changes directly. Under controlled conditions, these systems can recover speech with relatively high fidelity. However, they are highly sensitive to alignment, surface reflectivity, and environmental disturbances. Even small angular deviations can significantly degrade signal quality, limiting robustness in real-world scenarios.

#### 5.3.2. Rough-Focused and Speckle-Based Laser Microphones

To reduce alignment sensitivity, rough-focused laser microphones illuminate a larger surface area, producing speckle patterns rather than a single coherent reflection [39]. Speech information is extracted from temporal variations in the speckle field, often framed as an image-to-audio mapping task. This increased robustness comes at the cost of severe spectral degradation, including phase distortion and attenuation of high-frequency components. Early enhancement approaches primarily focused on reconstructing the magnitude spectrum, improving intelligibility while leaving significant perceptual artifacts [40].

#### 5.3.3. Phase-Aware and Learning-Based Enhancement

Recent advances incorporate phase-aware and complex-spectrum reconstruction techniques to address the limitations of amplitude-only enhancement [41]. By jointly estimating magnitude and phase, these methods yield improved perceptual quality compared to earlier approaches. In parallel, generative adversarial network (GAN)-based models have been proposed to extend the effective bandwidth of laser microphone signals, particularly in eavesdropping scenarios [42]. While such models can produce perceptually convincing speech, they often rely on hallucinated high-frequency content, raising concerns regarding physical fidelity and task-dependent reliability.

### 5.4. LiDAR-Derived Acoustic Sensing

LiDAR-based speech sensing repurposes time-of-flight or FMCW LiDAR systems, originally designed for ranging, to detect distance modulations caused by sound-induced vibrations [43]. Because these systems are not optimized for vibration sensing, their temporal resolution and sensitivity are limited. As a result, LiDAR-derived signals typically support only coarse speech-related tasks, such as digit recognition or keyword spotting, rather than full speech reconstruction [44]. Nevertheless, this line of work demonstrates that acoustic side-channel information can be extracted from a broad class of optical sensing systems.

### 5.5. Recent Frontiers in Optical Recovery (2025–2026)

The latest advancements in laser-based sensing focus on overcoming the mechanical damping of target materials and the persistent challenge of training data scarcity. Chen et al. (2025) [45] addressed the data bottleneck by developing a *physics-informed data augmentation strategy* that replicates material-specific distortions and laser speckle noise. By incorporating transient impulse noise to simulate laser interference patterns (speckle noise), they demonstrated that models trained on these physics-inspired simulations significantly enhance intelligibility across diverse surfaces, achieving a short-time objective intelligibility (STOI) score of 0.76 at 0 dB SNR.

Similarly, ref. [46] proposed a generative framework using *dual style encoders* to separately model high-frequency attenuation and noise characteristics inherent to optical microphones. This approach allows for the synthesis of large-scale degraded datasets from limited recorded samples, effectively modeling the non-linear degradation arising from the target object’s mass and stiffness. Their results confirmed that separate modeling of these degradation components effectively mitigates the effects of training data scarcity and improves perceptual quality.

To handle the structural complexity and long-range temporal dependencies of these signals, ref. [47] introduced a *Nested U-network with Gated Temporal Convolution (TCNUNet)*. The reasoning for the nested design is its ability to perform multi-stage refinement of material-specific distortions, thereby enabling better inversion of the complex, unknown transfer function between speech and the observed vibration signal. By employing gated temporal convolutions, the model adaptively suppresses transient artifacts and compensates for the non-stationary frequency smearing typical of real-world LDV data.

### 5.6. Discussion and Open Challenges

Across LDV, laser microphones, and LiDAR-based systems, a common trend is evident: learning-based methods increasingly compensate for fundamental physical limitations. While deep learning improves intelligibility and task performance, it cannot fully overcome constraints imposed by bandwidth, object dependence, and phase ambiguity. Key open challenges include robust cross-object generalization, physically grounded phase recovery, and the lack of standardized datasets for fair comparison. Addressing these challenges will likely require tighter integration of physical modeling and data-driven learning rather than purely end-to-end approaches.

### 5.7. The Modality Research Gap: Economic and Hardware Drivers

A notable trend in the literature is the disproportionate volume of research dedicated to mmWave radar compared to laser-based vibrometry. While laser Doppler vibrometry (LDV) offers superior physical precision and a higher signal-to-noise ratio (SNR) than radar under the same conditions, its adoption in the broader research community is constrained by high hardware costs and limited accessibility. Research-grade LDV systems typically range from $20,000 to over $100,000, effectively restricting their use to specialized acoustics and structural health monitoring laboratories.

In contrast, the surge in mmWave radar research is driven by the “commodity-scale” availability of the hardware. Due to the integration of 60 GHz and 77 GHz radar into automotive safety systems and consumer electronics (e.g., phones, healthcare monitors), high-performance single-chip radar modules are now available at a fraction of the cost (approximately $15–$50). This low barrier to entry has enabled a broader range of researchers to explore radar as an alternative to microphones. Consequently, while radar signals are physically more complex and “noisier” than laser reflections, the sheer volume of algorithmic innovation in the radar domain underscores its potential for large-scale, low-cost deployment in civilian and security applications.

## 6. Cross-Modality Comparison and Design Trade-Offs

Across the surveyed literature, system performance is reported using a diverse set of evaluation metrics, reflecting differences in sensing modalities, task definitions, and experimental protocols. Commonly used measures include Perceptual Evaluation of Speech Quality (PESQ), Short-Time Objective Intelligibility (STOI), Word Error Rate (WER), Character Error Rate (CER), Mean Opinion Score (MOS), and Segmental Signal-to-Noise Ratio (SegSNR) [48,49,50]. The comparative performance of these laser and radar based systems is summarized in Table 1 and Table 2.

### Typical Performance Ranges Across Task Complexities

To assess the practical maturity of vibration-based speech sensing without conflating results from mismatched experimental baselines, performance should be categorized by task complexity rather than by absolute metrics. Crucially, evaluating the starting baseline of the raw signals demonstrates that both radar and laser systems rely heavily on algorithmic enhancement to achieve practical results.

**1. Closed-Vocabulary Classification:**

For heavily constrained tasks such as digit recognition (e.g., AudioMNIST), radar-based systems achieve high maturity only after significant denoising. For instance, the raw, unprocessed signal in mmPhone [23] begins with severely degraded intelligibility (STOI ≈ 0.40) and a peak SNR of only ∼10 dB. Post-enhancement, *mmPhone* demonstrates >93% digit recognition accuracy through soundproof glass at 5 m [23]. Similarly, *mmSpy* achieves 83.33% accuracy at 0.3 m, though performance sharply degrades to 47.99% at 1.8 m [1].

Laser-based systems also demonstrate strong performance on constrained tasks when sufficient vibration information is preserved. For instance, in the *LidarPhone* study, the heavily bottlenecked audio recovered from a robot vacuum’s LiDAR is incapable of open-vocabulary transcription due to a low 1.8 kHz sampling rate. However, by processing the recovered spectrograms with a Convolutional Neural Network (CNN), the system successfully achieves a 91% accuracy for closed-vocabulary digit classification (0–9) and 96% for gender classification when targeting household objects at 1.5 m [43].

**2. Large-Vocabulary ASR:**

For unconstrained text transcription (e.g., 13,000+ words), radar performance limits are strictly dictated by sensing distance and physical algorithms, as raw mmWave signals are highly unintelligible. In *Radio2Text*, feeding the raw signals into a standard ASR network yields a baseline Word Error Rate (WER) of 45.1%. However, their tailored cross-modal architecture reduces this to a highly practical 9.4% WER at 0.5 m (which degrades to 15.4% at 1.5 m) [9]. Similarly, *mmSpeech* reports an initial catastrophic WER of 86.79% from the raw mmWave vibrations, which is algorithmically reconstructed and fine-tuned down to a 34.20% WER from 69.92 [2]. Without robust domain adaptation, attacks on unconstrained vocabularies yield high error rates, such as *mmWave-Whisper* achieving approximately 44.7% word accuracy at close ranges [10].

The lack of intelligible consonants in the recovered vibration signals heavily bottlenecks laser-based ASR performance. In the *Laser Meager Listener* study, distant eavesdropping on vibrating objects yields audio so degraded that feeding it into commercial ASR engines (e.g., Google Speech-to-Text) results in a starting point of near 0% to 17% maximum decoding accuracy [8].

**3. Speech Reconstruction Quality and Multi-Modal Robustness:**

For speech reconstruction tasks, laser-based systems are typically evaluated using perceptual speech quality metrics. Because the mechanical transfer function of vibrating objects naturally attenuates high frequencies, the raw optical signal often exhibits degraded fidelity. For instance, when an LDV targets an empty PET bottle from 3 m, the captured speech begins at only 1.76 PESQ and 0.85 STOI. After applying a two-stage waveform-based Deep Neural Network (DNN), the reconstructed signal improves to 2.35 PESQ and 0.94 STOI [54].

In radar-based robustness scenarios, advanced algorithms enable operation beyond direct line of sight. In Non-Line-of-Sight (NLoS) environments, multipath clustering algorithms dictate system survival. *AmbiEar* maintains a post-model WER of 16.19% in NLoS (compared to 15.01% in LoS), effectively rescuing the signal from a baseline direct-sensing approach that completely fails with an initial 95.92% WER [3].

Finally, multimodal systems that combine vibration sensing with microphones demonstrate the highest robustness in noisy environments. Under severe ambient noise, baseline audio-only and mmWave-only approaches suffer from WERs of 73.24% and 40.76%, respectively; however, post-fusion, *Wavoice* sustains a Character Error Rate (CER) below 1% at ranges up to 7 m [4]. Likewise, *RadioSES* demonstrates massive gains across different tasks: in single-speaker enhancement, it takes a noisy raw input with a baseline SiSDR of 3.9 dB and PESQ of 1.55, and pushes it to 14.5 dB SiSDR and a PESQ of 2.68. Furthermore, in complex multi-speaker separation tasks (e.g., 2-person noisy mixtures), it successfully elevates a severely degraded baseline of −1.7 dB SiSDR to 10.9 dB SiSDR [18].

Laser systems also benefit from multi-modal fusion despite requiring line-of-sight to the vibrating surface. Because LDV measurements are intrinsically immune to ambient acoustic noise, they can complement acoustic microphones. In a noisy moving-vehicle scenario, a standard acoustic microphone yields a baseline WER of 87.5%. By fusing this signal with an auxiliary LDV sensor for harmonic tracking, the system reduces the WER to 48.4% [35]. Similarly, when deep neural networks concatenate acoustic and LDV features, systems evaluated under noisy conditions reduce WER from 71.96% to 62.93%, and, when initialized on large-scale datasets, further reduce it from 32.93% to 25.22% WER [6].

## 7. Cross-Modality Comparative Analysis

Even when restricting comparison to a single modality, direct numerical benchmarking remains problematic due to significant environmental variance across experimental setups. This “Environmental Variance” acts as a hidden variable that sets the upper bound on achievable fidelity, regardless of the algorithm used.

In laser-based acoustic sensing, the mechanical transfer function and the target’s elasticity are the primary constraints. For instance, studies evaluate highly flexible membranes like PET bottles [37], paper coffee cups [8], and plastic trash bags [43], which yield vastly different acoustic responses compared to rigid surfaces like glass doors or human anatomical targets like the larynx [35].

Similarly, in radar-based sensing, signal quality depends on the target’s dielectric properties and the presence of complex multipath interference. Performance varies drastically between unobstructed Line-of-Sight (LoS) setups, through-wall scenarios [21], and Non-Line-of-Sight (NLoS) settings that rely on ambient reflections [3]. Furthermore, the operational distances in the literature range from centimeters [43] to tens of meters [55], introducing an exponential variance in the link budget and noise floor.

Because the majority of the literature lacks standardized reporting of physical metadata, such as absolute SNR at the sensor head, target material impedance, precise measurement distances, and geometric grazing angles, a quantitative meta-analysis risks attributing environmental advantages (such as a more reflective target or a shorter distance) to algorithmic superiority. Consequently, forcing a direct comparison between the performance metrics of these diverse systems, such as equating the Word Error Rate (WER) of an ASR model trained on NLoS ambient reflections with the PESQ of a reconstruction model tested on a smartphone chassis at 30 cm, is methodologically unsound. While structured summary tables provide a macroscopic view of the state of the art, the field lacks unified benchmark datasets and standardized reporting protocols required for rigorous numerical comparisons.

Despite these experimental heterogeneities, radar and laser sensing operate on different electromagnetic principles. Their shared status unifies them as an *inverse problem*. In both modalities, the goal is to recover a latent acoustic signal x(t) from a distorted observation y(t)=F(x,M,E)+n, where *M* represents the mechanical impedance of the target and *E* represents the geometric environmental constraints and *n* represents the additive noise.

Our analysis reveals that the convergence toward similar deep-learning architectures is a direct response to these shared physical constraints. (Modality hardware comparison can be found in Table 3.) However, the internal configuration of these models is parametrically tuned to the specific sensing physics:

- **Generative Priors:** Radar-based recovery relies on more aggressive generative priors (e.g., Diffusion or GANs) to bridge the significant modality gap between low-resolution RF returns and broadband speech. Laser-based systems, which offer higher raw fidelity, primarily use these models for spectral denoising and harmonic restoration.
- **Temporal Attention:** Both modalities utilize Self-Attention (Transformers) but for different physical reasons. In radar, attention mechanisms provide robustness against multipath-induced signal dropouts, while in laser sensing, they compensate for non-stationary frequency smearing caused by target surface heterogeneity.

This side-by-side analysis suggests that the unification of radar and laser speech sensing extends beyond a mere structural choice; it represents a methodological alignment driven by the underlying physics of vibration recovery.

## 8. Open Challenges and Research Directions

Data scarcity remains the most fundamental bottleneck in radar- and laser-based speech sensing. To date, there are no publicly available, standardized datasets for speech reconstruction or automatic speech recognition (ASR) using mmWave radar or laser-based modalities. Existing studies rely almost exclusively on private, small-scale datasets collected under tightly controlled and highly specific conditions, often tailored to a particular sensing setup, target object, distance, material, or geometry. This lack of open, standardized data severely limits reproducibility, prevents fair cross-paper comparisons, and hinders systematic evaluation of generalization across environments and sensing configurations.

As a result, community-scale benchmarking remains in its infancy. Recent initiatives such as the Radar-based Acoustic Sensing and Eavesdropping (RASE) challenge represent an important step toward shared tasks, datasets, and evaluation protocols. However, the outcomes of these efforts have not yet been fully reported, and the data released to date consist only of reconstructed audio waveforms, not raw radar or optical sensing signals. Moreover, the overall scale of the dataset (on the order of ∼10 h) remains limited relative to the data requirements of modern data-hungry learning frameworks, particularly for end-to-end speech reconstruction, robust ASR, or cross-domain generalization studies. Until larger, modality-native, and openly accessible benchmarks emerge, it remains difficult to quantify progress or establish performance baselines comparable to those used in conventional noisy-speech and audio-only research.

Beyond data availability, a major open challenge lies in generalization across physical configurations. Current radar- and laser-based systems are highly sensitive to sensing direction, distance, object geometry, surface material, and multipath effects. While promising results are often demonstrated in fixed or carefully aligned setups, performance typically degrades when these conditions change. Achieving plug-and-play deployment in real-world scenarios, therefore, requires models that generalize across a broad range of orientations, distances, and environments without scene-specific calibration.

From a speech reconstruction perspective, the absence of high-frequency content remains a fundamental limitation. Both radar and laser modalities sense speech indirectly via mechanically induced vibrations, resulting in significant attenuation of high-frequency components critical to articulation, timbre, and speaker characteristics. Consequently, reconstructed signals often exhibit an unnatural tone, reduced clarity, and limited vocal realism. Addressing this limitation requires tighter integration of physical modeling with learning-based bandwidth extension, rather than relying solely on data-driven hallucination.

For automatic speech recognition (ASR), current radar- and laser-based systems remain constrained in vocabulary size, linguistic richness, and contextual modeling. Although recent work has progressed beyond keyword spotting toward open-vocabulary transcription, error rates remain substantially higher than those obtained from noisy acoustic speech. Generalizing to multiple languages, longer utterances, richer linguistic contexts, and increased sensing distances remains an open research problem.

Several promising research directions emerge from these limitations. One is the use of large pretrained audio foundation models to guide radar or laser encoders in mimicking speech characteristics through cross-modal or multi-level knowledge distillation. Another is physics-aware learning, which explicitly models where physical sensing fails, particularly at higher frequencies, and compensates for these losses in a principled manner. Exploiting multiple objects and multipath diversity may further improve robustness by leveraging redundant vibration sources present in realistic environments.

Finally, achieving perceptually realistic speech, including natural prosody, voicing, and speaker identity, remains largely unsolved. Progress toward this goal will require advances in sensing hardware, physical modeling, multimodal learning, and critically, the release of open, diverse, and standardized datasets, reinforcing that radar- and laser-based speech sensing is an open and evolving research field rather than a closed or mature technology.

## 9. Ethical Considerations and Privacy Implications

As the fidelity of non-acoustic speech recovery approaches that of conventional microphones, the ethical implications of this technology necessitate careful consideration. The “dual-use” nature of vibration-based sensing presents a significant tension between transformative beneficial applications and inherent privacy risks. Unlike traditional acoustic sensing, which can be mitigated by soundproofing or acoustic dampening, vibration-based recovery can bypass physical barriers such as soundproof glass or heavy doors. This enables the potential for covert eavesdropping on private conversations without the subject’s knowledge or consent, posing a challenge to established norms of physical privacy.

However, these modalities offer legitimate and vital advantages for speech enhancement and accessibility. Laser and radar systems can facilitate high-quality speech capture from a distance, serving as a powerful tool for emergency response, search-and-rescue operations, or assistive hearing in extremely noisy industrial environments where traditional microphones fail. These systems enable “hearing from afar” and communication through barriers without requiring the speaker to increase vocal effort, which is particularly beneficial for low-power edge devices and for hands-free communication in sterile or hazardous environments.

Ultimately, the trajectory of this field depends on its responsible usage. We believe that as this research area grows, it must be accompanied by a rigorous understanding of ethical deployment. Future work should prioritize “privacy-by-design” frameworks, including the development of physical perturbation techniques [19], such as targeted surface-vibration interference, to jam unauthorized sensing while maintaining the integrity of legitimate systems. By establishing ethical guidelines for data collection and recognizing the sovereignty of personal acoustic spaces, the field of vibration-based sensing can continue to evolve as a beneficial pillar of modern signal processing.

## 10. Conclusions

Non-acoustic speech sensing has progressed from early proof-of-concept vibrometry toward learning-based systems capable of intelligible reconstruction and, in some cases, large-vocabulary recognition. Across mmWave radar and laser/LDV/LiDAR families, the literature shows consistent trade-offs: radar offers broader deployability and potential NLoS operation. Still, it is fundamentally bandwidth-limited, whereas optical vibrometry can preserve finer vibration detail but typically requires line-of-sight and careful alignment. Despite rapid model-driven gains, current performance remains strongly dependent on target surface properties, scene geometry, and motion artifacts, and results are difficult to compare due to the lack of standardized datasets and protocols. Near-term progress is likely to come from (i) benchmark-quality shared datasets that include raw sensor signals, (ii) physics-aware learning that explicitly models sensing bandwidth limits and multipath/speckle failure modes, and (iii) principled cross-modal training strategies (distillation, self-supervision, and synthetic-to-real adaptation) that reduce reliance on tightly controlled private collections. Ultimately, making these systems robust across environments and targets is the key step required before claims of general-purpose “speech from vibrations” can be evaluated on the same footing as conventional speech enhancement and ASR.

## Figures and Tables

**Table 1:** Summary of laser microphone, LDV, and LiDAR-based systems for sound recovery and speech recognition.

Paper	Mission	Sensor Type	Setup	Model/Method	Results
The novel role of arctangent phase algorithm and voice enhancement techniques in laser hearing [51]	Speech enhancement	LDV (532 nm, 50 mW)	50–75 m; human throat	Quad. demodulation, Wiener filtering, MCRA	MOS: 3.4 → 4.0; SNR: 11.14dB
Audio Signal Extraction and Enhancement Based on CNN From Laser Speckles [39]	Speech enhancement	Green laser (532 nm, 50 mW)	∼5 m; speaker diaphragm	16-layer CNN on laser speckle patterns	SegSNR: +4.9 dB; LLR: +8.9%
Deep Learning-Based Speech Enhancement with Rough-Focused Optical Laser Microphone by Reconstructing Complex Spectrum [41]	Robust speech enhancement	LDV (Polytec VFX-F-110), Laser microphone	Reflective surfaces; 1, 60, 120, 180 mm spot	DCCRN; complex-spectrum reconstruction	Improved PESQ from raw audio ∼2.1 to ∼2.9; better phase recovery
Deep Neural Network for Robust Speech Recognition with Auxiliary Features from Laser-Doppler Vibrometer Sensor [6]	Robust ASR	LDV (VocalZoom, 780 nm) and microphone	measuring the human throat/larynx	Directed at throat; close range, DNN regression; DNN-HMM ASR (LMFB)	WER reduced by ∼6% vs. acoustic
Speech Measurements Using a Laser Doppler Vibrometer Sensor: Application to Speech Enhancement [52]	Enhancement and VAD	LDV (VibroMet 500 V, 780 nm)	75 cm; human throat/larynx	Kurtosis speckle filtering, TF-VAD, OM-LSA	segSNR 7.64
Speech Enhancement Based on Two-Stage Processing [37]	Distant-talk Speech Enhancement	LDV	Objects: PET bottle, cardboard, Al-sheet, plastic.	Two-Stage DNN: 1. STFT-based denoising/phase estimation. 2. Waveform-based LF/HF restoration.	Cosine Distance reduced by ≈0.4 (0–4 kHz) vs. GLA. Improved intelligibility.
Laser Meager Listener: A Scientific Exploration of Laser-based Speech Eavesdropping [8]	Eavesdropping, recognition	LDV (Polytec PDV-100)	Paper, plastic; variable dist.	Time/freq analysis, cross-correlation, ssubmmse	STOI: 0.39; PESQ: 1.50
Spying with Your Robot Vacuum Cleaner: Eavesdropping via Lidar Sensors (LidarPhone) [43]	Eavesdropping	LiDAR (Xiaomi Roborock S5)	Up to 150 cm; household objects	Filtering, spectral denoising, CNN classifier	Digit: ∼91%; Gender: ∼96%
Speech Enhancement for Optical Laser Microphone with Deep Neural Network [37]	Speech enhancement	LDV (NLV-2500-5)	3 m; empty PET bottle	Two-stage DNN: ASE (UNet) + PSE (TCNN)	PESQ: ∼1.96; STOI: ∼0.85
Spectrogram-based Speech Enhancement by Spatial Attention Generative Adversarial Networks [53]	Speech reconstruction	LDV-based laser listening	1 m; glass cabinet door	SAGAN-stoi (GAN with spatial attention, STFT/ISTFT)	PESQ: 1.565; STOI: 0.747

**Table 2:** Summary of mmWave radar-based systems for sound recovery, speech recognition, and eavesdropping.

Paper	Mission	Radar Type	Setup	Model/Method	Results
mmSpy: Eavesdropping on phone calls via earpiece vibration [1]	Digit classification/keyword recognition	mmWave (60/77 GHz)	Radar aimed at phone back ((0.3 m–1.8 m); earpiece vibration sensing. Effective audio bandwidth up to 4 kHz.	CNN-based classifier with autoencoder-based enhancement	Up to 83% digit accuracy; accuracy degrades with distance
MILLIEAR: Acoustic eavesdropping with unconstrained vocabulary [5]	Audio reconstruction for eavesdropping	mmWave FMCW (77–81 GHZ)	Speech sensed through insulating materials (glass, plastic, paper). resolution audio bandwidth up to 22.05 kHz.	cGAN-based audio spectrogram reconstruction	MCD ≈ 3.68; effective on unconstrained vocabulary
RadioMic: Sound sensing via mmWave signals [17]	Audio reconstruction	mmWave 77 GHz with a bandwidth of 3.52 GHz	Through-wall sensing(up to 4 m active, 2 m passive); active (throat, speaker) and passive objects. Captures speech bandwidth up to 3.125 kHz; RF Bandwidth: 3.52 GHz	Radio acoustics processing with neural enhancement and antenna diversity	Audio recovered through walls; SNR/PESQ depend on LoS/NLoS and source
Radio2Speech: Recover high-quality speech in noisy scenarios [28]	Speech recovery	mmWave FMCW (77–81 GHz)	0.3–0.5 m from loudspeaker; soundproof and noisy rooms. Captures an effective audio bandwidth of 60–4000 Hz.	Radio UNet with Transformer encoder + Parallel WaveGAN	STOI ∼0.89; PESQ ∼2.50 in quiet conditions
mmSpeech: End-to-end eavesdropping via vibrating objects [2]	Speech reconstruction for eavesdropping	mmWave (60–64 GHz)	Passive vibrating objects like PET film, foil(0.5 m–4.5 m); LoS sensing. Speech bandwidth limited to <4 kHz.	End-to-end DNN trained on synthetic radar–audio data	STOI ∼0.80 (PET); FWSegSNR ∼9.43 dB
mmPhone: Eavesdropping on loudspeakers inside soundproof rooms [23]	Eavesdropping	mmWave FMCW (77–81 GHz)	Piezoelectric film interrogation through soundproof walls (0.8 m up to 5 m). Recovers wideband audio up to 5.1 kHz via Harmonic Extension.	DNN denoising with multi-channel phase alignment	>93% digit accuracy at 5 m
mmProcess: Phase-Based Speech Reconstruction from mmWave Radar [32]	Speech reconstruction	mmWave radar (76–81 GHz)	Fine vibration sensing of objects in range (approx. 25 cm)	Signal processing only (range FFT, peak tracking, phase filtering)	Intelligible speech without learning; sensitive to object and environment
Radio2Text: Real-time streaming ASR with large vocabulary [9]	Streaming ASR	mmWave radar (77–81 Ghz)	Near loudspeaker; quiet, noisy, and soundproof environments. (50 cm–150 cm). Covers human speech bandwidth up to 8 kHz.	Streaming Transformer ASR with cross-modal knowledge distillation	5.7% CER; 9.4% WER in clean conditions; low latency
mmWave-Whisper: Phone call eavesdropping and transcription [10]	Eavesdropping/ ASR	mmWave (77–81 GHz)	Radar aimed at phone back (25–125 cm)	LoRA-adapted Whisper-large-v2 trained on synthetic radar–audio pairs	44.74% word accuracy; 62.52% character accuracy
WaVoice: Noise-resistant multi-modal speech recognition [4]	Multi-modal ASR	mmWave radar (77–81 GHz) + microphone	Office, café, subway; range up to 7 m. Mel-filter banks cover a frequency bandwidth up to 8 kHz.	Attention-based audio–radar fusion with coherent demodulation	CER < 1% within 7 m under controlled conditions
mmMIC: Multi-modal speech recognition [29]	Multi-modal ASR	mmWave (60–64 GHz)	Vocal-cord vibration sensing with visual articulatory cues. (0.5 m–3.0 m)	Attention-based multi-modal fusion	92.8% average recognition accuracy
RadioSES: Audio–radio speech enhancement and separation [18]	Speech enhancement/ separation	mmWave radar (77 GHz with 3.52 GHz BW) + microphone	Multi-speaker, noisy environments. (approx. 40 cm)	RadioSESNet with DPRNN; radar-based VAD and pitch estimation	3 to 6 dB SiSDR improvements in separating two and three-person mixtures, respectively.
AmbiEar: Voice recognition in non-line-of-sight scenarios [3]	NLoS ASR	mmWave (77–81 GHz)	Ambient object vibrations sensed via multipath reflections	Signal superimposition with encoder–decoder recognition network	87.21% word accuracy; 35% error reduction vs. direct sensing
EveGuard: Defeating vibration-based side-channel eavesdropping [19]	Defense/ countermeasure	mmWave radar (76–81 GHz), LDV, inertial sensors	Protection against optical, laser, and radar-based eavesdropping. (0.5 m–1.5 m sensor-to-object; up to 12 m remote attack)	Eve-GAN with perturbation generation model	WER increased from 12.2% to >73.2%

**Table 3:** High-level comparison between mmWave radar- and laser/LDV-based speech sensing.

Property	mmWave Radar	Laser/LDV/LiDAR
Line-of-sight (LoS) requirement	Optional (can exploit multipath/NLoS)	Typically required (target surface visibility)
Typical operational distance	Medium–long (setup-dependent)	Long for reflective targets; often alignment-limited
High-frequency recovery potential	Poor–moderate (bandwidth-limited; relies on priors)	Moderate–good (surface vibration sensing can preserve more detail)
Sensitivity to motion	High (phase noise, multipath, micro-motions)	High (speckle, pointing jitter; but different failure modes)
Target dependence	Strong (material/geometry/vibration quality)	Strong (reflectivity, surface roughness, angle)
Hardware cost	Low–medium (commodity mmWave exists)	Medium–high (LDV expensive; LiDAR varies)
Stealth/detectability	High	Low–medium (visible beam risk; IR depends)
Consumer viability	High (radars in devices)	Low–medium (LiDAR in some devices; LDV not)

## Data Availability

The open-source data mentioned in this paper, which serves as the foundation for the proposed standardized dataset requirements, is based on the Radar-based Acoustic Sensing and Eavesdropping (RASE) Challenge dataset. This dataset consists of pre-processed waveforms collected via a TI AWR2243BOOST mmWave radar. The dataset is accessible at: https://rase-challenge.github.io/RASE2026-Challenge/ (accessed on 4 March 2026).
